# Obesity and Pregnancy: Impact on Childbirth Timing, Delivery Mode, and Maternal Recovery: An Update

**DOI:** 10.3390/medsci13030182

**Published:** 2025-09-10

**Authors:** Angeliki Gerede, Maria Danavasi, Sofoklis Stavros, Anastasios Potiris, Athanasios Zikopoulos, Efthalia Moustakli, Charikleia Skentou, Ekaterini Domali, Nikolaos Nikolettos, Makarios Eleftheriades

**Affiliations:** 1 Department of Obstetrics and Gynecology, Democritus University of Thrace, 69100 Alexandroupolis, Greece; mairidanavasi4800@gmail.com (M.D.); nnikolet@med.duth.gr (N.N.); 2 Third Department of Obstetrics and Gynecology, University General Hospital “ATTIKON”, Medical School, National and Kapodistrian University of Athens, 12462 Athens, Greece; sfstavrou@med.uoa.gr (S.S.); apotiris@med.uoa.gr (A.P.); thanzik92@gmail.com (A.Z.); 3 Laboratory of Medical Genetics, Faculty of Medicine, School of Health Sciences, University of Ioannina, 45110 Ioannina, Greece; thaleia.moustakli@gmail.com; 4 Department of Ostetrics and Gynecology, Medical School of the University of Ioannina, 45110 Ioannina, Greece; haraskentou@uoi.gr; 5 First Department of Obstetrics and Gynecology, Alexandra Hospital, Medical School, National and Kapodistrian University of Athens, 11528 Athens, Greece; kdomali@yahoo.fr; 6 Second Department of Obstetrics and Gynecology, University Hospital “Aretaieion”, Medical School, National and Kapodistrian University of Athens, 11528 Athens, Greece; melefth@med.uoa.gr

**Keywords:** maternal obesity, pregnancy outcomes, cesarean delivery, macrosomia, metabolic disorders, maternal health

## Abstract

This review explores the impact of maternal obesity on pregnancy outcomes, emphasizing its significant global health challenge and profound implications for both mothers and infants. It influences the timing and mode of childbirth, elevating the risk of conditions like hypertensive disorders, cesarean delivery, and gestational diabetes mellitus. The review focuses on analyzing how maternal obesity affects postpartum recovery, birth timing, and delivery methods. Relevant studies were identified using PubMed and Scopus. Findings indicate that obese pregnant women are at higher risk for medically indicated preterm birth, scheduled and emergency cesarean sections, and labor induction. Postpartum recovery is often prolonged due to breastfeeding challenges, infection risks, and delayed wound healing. Additionally, maternal obesity increases the likelihood of fetal complications such as macrosomia and long-term metabolic disorders. These results highlight the importance of personalized treatments and early weight control to improve the health of both mother and baby. A comprehensive approach integrating clinical care, public health initiatives, and policy measures is essential to reduce pregnancy complications associated with obesity.

## 1. Introduction

Obesity has become a major public health issue, particularly in Western countries. Its prevalence among pregnant women has risen globally [1,2]. Up to 30% of pregnant women in high-income countries are classified as obese, and approximately 40% exceed the recommended gestational weight gain (GWG) [3]. Recent studies and meta-analyses suggest that maternal obesity and excessive GWG can lead to long-term effects on offspring adiposity, cardiovascular, and respiratory health [3,4,5].

The increasing rates of obesity among reproductive-aged women raise concerns for both maternal and child health. Understanding how obesity affects pregnancy and childbirth is essential to develop effective clinical interventions and shape public health policies [6,7,8,9].

Maternal obesity alters hormonal balance, cardiovascular function, metabolism, and immune responses [3]. It increases the risk of gestational diabetes mellitus (GDM), hypertensive disorders, preeclampsia, and non-alcoholic fatty liver disease (NAFLD), all of which contribute to adverse maternal and neonatal outcomes [10]. It also impairs placental function, disrupts fetal growth, and predisposes children to long-term metabolic and cardiovascular diseases [10]. Excessive GWG further contributes to postpartum complications, reduced breastfeeding, and childhood obesity [11,12].

Obesity during pregnancy also affects neonatal outcomes. It is linked to fetal macrosomia, preterm birth, congenital anomalies, and stillbirth [3]. The concept of “fetal programming” suggests that intrauterine exposure to maternal obesity may predispose offspring to obesity, metabolic syndrome, and cardiovascular disease later in life [4].

In addition, maternal obesity may alter breast milk composition, including macronutrient content and microbiota, influencing infant feeding behavior and early metabolic regulation [5]. Recent studies also associate maternal obesity with placental inflammation, gut microbiome disruption, and oxidative stress, which may further compromise pregnancy outcomes [13].

Given the growing prevalence of maternal obesity, early intervention is crucial. This review synthesizes current evidence on how maternal obesity affects childbirth—specifically, timing, mode of delivery, and postpartum recovery—to inform clinical decisions and public health strategies aimed at improving outcomes for mothers and their children [14].

## 2. Materials and Methods

This narrative review was conducted based on a structured literature search across two primary databases: PubMed and Scopus. The goal was to identify peer-reviewed studies examining the impact of maternal obesity on pregnancy-related outcomes, focusing on childbirth timing, delivery mode, postpartum recovery, and neonatal complications.

The search strategy used both MeSH terms and free-text keywords, including “maternal obesity”, “gestational weight gain”, “pregnancy complications”, “cesarean delivery”, “labor induction”, “postpartum recovery”, “macrosomia”, “gestational diabetes”, and “placental dysfunction”. Boolean operators (AND, OR) were used to combine concepts and refine the results.

The search was limited to English-language articles published between 1 January 2018 and 31 March 2025. Earlier high-impact or foundational studies (e.g., Catalano et al., 2017) were also included to provide historical or conceptual context.

Included articles were original research (observational or interventional), systematic or narrative reviews, and meta-analyses relevant to maternal obesity and pregnancy outcomes. Excluded were case reports, conference abstracts, and studies focused solely on animal models or non-obstetric populations.

**Figure 1:**
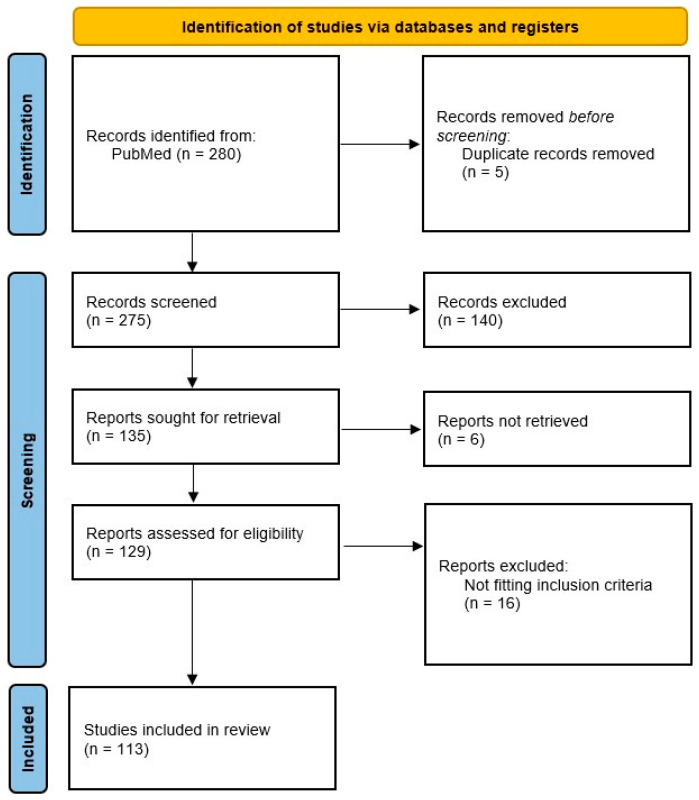
A PRISMA-like flowchart illustrating the article selection process.

The selection process followed PRISMA guidelines. After deduplication, titles and abstracts were screened for relevance. Full-text eligibility was then assessed by two independent reviewers. Discrepancies were resolved by discussion. A PRISMA-like flowchart illustrating the article selection process is provided in Figure 1.

The findings were synthesized narratively to highlight current knowledge, identify research gaps, and inform future clinical and public health practices.

## 3. Maternal Obesity and Pregnancy Complications

### 3.1. Pathophysiology of Obesity During Pregnancy

Maternal obesity triggers a complex cascade of physiological and molecular alterations that disrupt normal pregnancy processes and increase the risk of adverse outcomes. These alterations include chronic low-grade inflammation, insulin resistance, oxidative stress, hormonal imbalances, placental dysfunction, and epigenetic modifications [15,16].

Obesity is characterized by systemic inflammation driven by excessive adipose tissue, which functions as an active endocrine organ. Adipocytes secrete pro-inflammatory cytokines such as TNF-α, IL-6, and CRP, which interfere with immunological tolerance during pregnancy, promote oxidative stress, and impair endothelial nitric oxide (NO) production—hallmarks of preeclampsia and gestational diabetes mellitus (GDM) [17,18,19,20].

Adipose tissue also modulates metabolic homeostasis through hormonal secretions. Elevated leptin levels impair hypothalamic appetite regulation and energy balance, contributing to excessive gestational weight gain (GWG), while reduced adiponectin impairs glucose utilization and enhances systemic inflammation, increasing susceptibility to GDM [21,22].

At the placental level, maternal obesity leads to increased expression of glucose (GLUT1) and lipid (FATP) transporters, enhancing nutrient transfer and predisposing to fetal overgrowth and macrosomia [23]. Concurrently, elevated reactive oxygen species (ROS) impair trophoblast function and angiogenesis, increasing the risk of intrauterine growth restriction (IUGR) and stillbirth [24].

Epigenetic modifications constitute another pathway by which maternal obesity affects fetal development. Aberrant DNA methylation, histone modification, and dysregulated microRNA expression in the placenta and fetal tissues can program metabolic dysfunctions, increasing offspring risk for obesity and type 2 diabetes later in life [25].

Obesity also impacts labor physiology. Reduced expression of oxytocin receptors and connexin 43 in myometrial cells weakens uterine contractility, increasing the likelihood of labor dystocia and cesarean delivery [26].

Postpartum, obesity-related inflammation impairs wound healing via elevated matrix metalloproteinase (MMP) activity, while hormonal imbalances and insulin resistance hinder prolactin signaling and delay lactation [27].

Understanding these intertwined mechanisms reveals potential targets for intervention. Strategies focused on reducing inflammation, restoring hormonal balance, and reversing epigenetic dysregulation may improve both maternal and fetal outcomes [28]. Various molecular pathways contribute to pregnancy complications associated with obesity, including chronic inflammation, hormonal dysregulation, and placental dysfunction (Table 1).

### 3.2. Chronic Inflammation and Placental Dysfunction

Obesity-related adipose tissue secrets pro-inflammatory cytokines such as tumor necrosis factor-alpha (TNF-α), interleukin-6 (IL-6), and C-reactive protein (CRP) [14,29]. These cytokines impair placental angiogenesis and nutrient exchange, raising the risk of preeclampsia, fetal growth restriction, and preterm birth.

Oxidative stress, a key mechanism, arises from excess adiposity and overwhelms antioxidant defenses in the placenta [30]. This damages trophoblasts, reduces vascularization, and limits perfusion, increasing the risk of fetal hypoxia and intrauterine growth restriction (IUGR) [31,32]. Mitochondrial dysfunction further compromises placental nutrient transport and fosters lipid peroxidation and endothelial damage [1].

Deficiencies in glutathione peroxidase, superoxide dismutase, and catalase exacerbate oxidative stress and impair placental development [31]. This imbalance exacerbates oxidative stress, further impairing placental angiogenesis and increasing the risk of adverse pregnancy outcomes. Studies indicate that targeted antioxidant interventions may help mitigate oxidative damage, potentially reducing the risks associated with maternal obesity, preterm birth, and placental insufficiency [31]. Antioxidant therapies may mitigate this damage and reduce risks of preterm birth and placental insufficiency.

### 3.3. Insulin Resistance, Dyslipidemia, and Gestational Diabetes

Obesity heightens insulin resistance beyond physiological levels seen in pregnancy, predisposing to gestational diabetes mellitus (GDM) and related complications such as fetal macrosomia [33,34,35,36]. Additionally, obese pregnancies often exhibit dyslipidemia—elevated TGs, FFAs, and LDL/VLDL—which disrupt glucose metabolism and promote inflammation [37]. Placental lipid accumulation exacerbates oxidative stress and impairs fetal nutrient transfer [38]. This environment increases offspring risk of obesity, NAFLD, and metabolic syndrome [39].

### 3.4. Renin–Angiotensin System Dysregulation in Pregnancy

Obesity disrupts the renin–angiotensin system (RAS), intensifying vasoconstriction, inflammation, and endothelial dysfunction. Angiotensin II (Ang II), the key effector of RAS, is elevated in obese pregnancies and acts through AT1 receptors to promote vasoconstriction, oxidative stress, and pro-inflammatory cytokine release. These changes impair placental perfusion and contribute directly to hypertensive disorders such as preeclampsia [40,41]. In obese women, the overactivation of Ang II signaling is compounded by increased secretion of adipose-derived angiotensinogen, amplifying the systemic hypertensive burden [42,43,44]. This upregulation perpetuates a feedback loop of vascular stress, endothelial damage, and placental hypoxia.

Mechanistically, placental ischemia—common in obese pregnancies—triggers the release of bioactive factors including AT1 receptor agonistic autoantibodies (AT1-AAs), which further sensitize the vasculature to Ang II, intensifying vasoconstriction and inflammation [40]. These autoantibodies enhance calcium influx in vascular smooth muscle cells, promoting hypercontractility and impairing vascular adaptation to pregnancy demands.

### 3.5. Cardiovascular and Hemodynamic Stress in Obese Pregnancies

Pregnancy necessitates profound cardiovascular adaptations—including increased cardiac output, blood volume expansion, and systemic vasodilation—to support fetal development. In women with obesity, these changes are pathologically exaggerated, leading to hemodynamic overload and vascular dysfunction [45].

Obesity augments left ventricular workload and impaired myocardial efficiency, predisposing to subclinical cardiac remodeling and diastolic dysfunction [32]. Simultaneously, vascular compliance is reduced, and endothelial reactivity is compromised due to chronic low-grade inflammation and oxidative stress, which suppress nitric oxide bioavailability [1].

This dysfunctional hemodynamic state contributes to a heightened risk of pregnancy-induced hypertension (PIH), preeclampsia, and reduced placental perfusion. Elevated levels of circulating leptin and pro-inflammatory cytokines in obese pregnancies further exacerbate sympathetic activation, vascular tone, and systemic inflammation [46].

Recent studies demonstrate that these changes also promote a prothrombotic vascular phenotype, marked by increased expression of adhesion molecules, von Willebrand factor, and tissue factor, increasing the likelihood of thromboembolic events and endothelial activation [47].

Moreover, when combined with obesity-induced insulin resistance and metabolic dysregulation, the cumulative cardiovascular burden disrupts uteroplacental circulation, increasing the risk of preeclampsia, fetal growth restriction, and preterm birth [48].

Together, these findings underscore that maternal obesity imposes a dual burden—hemodynamic and metabolic—on the cardiovascular system, exacerbating maternal risk while compromising fetal oxygenation and nutrient delivery.

### 3.6. Microbiome Dysbiosis and Inflammation

Obese pregnancies show gut microbiota dysbiosis—lower microbial diversity and a higher Firmicutes-to-Bacteroidetes ratio—linked to insulin resistance, lipid imbalance, and systemic inflammation [49]. LPS-induced inflammation contributes to GDM and preeclampsia. Maternal microbiota also shapes fetal colonization, potentially programming obesity and immune dysregulation in offspring [50,51].

## 4. The Impact of Obesity on Childbirth Timing

Pregnancy outcomes are impacted by maternal obesity, which also has an impact on postpartum recovery, the delivery method, and the time of labor [52]. Pregnant women who are obese are more likely to experience postpartum problems, cesarean sections, and early labor induction [5]. Neonatal problems, including macrosomia and metabolic abnormalities, are also a result of maternal fat. Improving pregnancy outcomes for obese women and their children requires early maternal weight management interventions and tailored public health policies [36].

Obesity has emerged as a significant global health issue, particularly for fertile women. Rising obesity rates are associated with gestational diabetes, hypertensive diseases, preeclampsia, and cesarean delivery complications. Understanding the relationship between maternal obesity and pregnancy outcomes is essential to maximizing the health of both mothers and neonates. This review examines the effects of obesity on the mother’s recovery, the delivery technique, and the timing of labor [32]. In obese pregnancies, labor is often medically managed due to GDM, hypertensive disorders, and macrosomia, all of which increase the likelihood of labor induction and cesarean delivery [53,54]. The altered inflammatory and metabolic state in obesity contributes to delayed cervical ripening, dysfunctional labor, and an increased likelihood of failed labor induction. Furthermore, placental dysfunction, oxidative stress, and insulin resistance play crucial roles in altering fetal growth patterns, predisposing infants to macrosomia or IUGR [55].

### 4.1. Mode of Delivery in Obese Women

Maternal obesity is strongly associated with elevated cesarean delivery rates, with reports indicating 50–60% in obese women compared to 20–30% in those with a normal BMI [56]. Cesarean sections are often indicated due to fetal macrosomia, labor dystocia, comorbidities, and failed inductions. The mechanical and metabolic burdens of obesity impair uterine contractility and contribute to labor complications.

Although cesarean delivery may mitigate certain intrapartum risks, it introduces complications such as postpartum hemorrhage and surgical site infections. Obese women experience increased uterine mass and reduced myometrial responsiveness, necessitating aggressive postpartum management [57]. Delayed wound healing and infection risks are heightened by excessive adiposity [1].

Intraoperative challenges include surgical exposure difficulties, extended operative times, and anesthesia-related complications. Risks such as hypoxia and difficult intubation are higher in obese patients [58]. Furthermore, repeat cesareans in obese women carry a greater risk of uterine rupture due to increased intra-abdominal pressure and inflammatory responses, underscoring the importance of evaluating the candidacy for vaginal birth after cesarean (VBAC).

Although VBAC remains an option, it is associated with lower success rates and higher risks in obese women, particularly those with comorbidities like GDM and hypertension [32,59]. Proper candidate selection and comprehensive counseling are essential to weigh potential benefits and risks [60].

**Figure 2:**
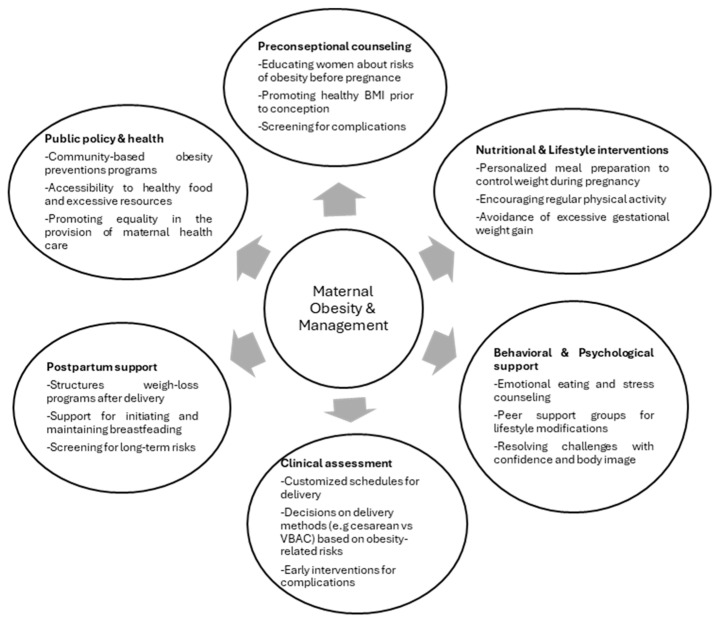
Comprehensive approach to maternal obesity management. This figure outlines key strategies for managing maternal obesity across various stages, including preconception, pregnancy, delivery, and postpartum care.

While VBAC could seem like a viable way to reduce cesarean deliveries, decisions should be individualized, considering each patient’s specific obesity-related risk factors to optimize maternal and fetal outcomes. A comprehensive approach to maternal obesity management involves multiple strategies, including preconception counseling, nutritional interventions, and postpartum support (Figure 2).

Obese women are more likely to experience labor dystocia, which is defined by irregular labor progression, because of increased fetal size, decreased uterine contractility, and excess soft tissue in the birth canal. A birth weight of more than 4000–4500 g is known as fetal macrosomia, and it is more common in pregnancies where the mother is obese. Shoulder dystocia, birth trauma, and a higher risk of needing an emergency cesarean section or an operative vaginal delivery are only a few of the major delivery issues that macrosomic babies present. To reduce the dangers to the mother and the newborn, these issues require careful fetal monitoring during labor and, frequently, early intervention [61].

### 4.2. Anesthesia and Surgical Considerations

Administering anesthesia in obese pregnancies presents considerable challenges. Physiological changes such as increased oxygen demand and decreased lung compliance elevate perioperative risks. Neuraxial anesthesia is preferred but technically more difficult due to anatomical distortion, often requiring ultrasound guidance [62,63].

General anesthesia carries higher risks of failed intubation, aspiration, and respiratory compromise. Altered pharmacokinetics in obesity prolong drug clearance, necessitating individualized anesthetic strategies. Multimodal analgesia is recommended to optimize pain control while minimizing respiratory depression [62].

Surgical risks include prolonged operative time, greater blood loss, and elevated infection rates due to impaired healing. Obese women are more susceptible to postpartum hemorrhage and thromboembolic events. Standard preventive strategies such as early mobilization and tailored thromboprophylaxis are essential [58]. Prophylactic strategies, such as preoperative weight-based antibiotic dosing, negative-pressure wound therapy, and subcutaneous drain placement, have been proposed to mitigate postoperative complications [62].

A multidisciplinary approach involving obstetricians, anesthesiologists, and maternal–fetal medicine specialists is vital to optimizing outcomes. Preoperative planning and postoperative surveillance can reduce maternal morbidity and improve neonatal health. Future research should refine anesthetic protocols and develop enhanced monitoring to address obesity-related surgical challenges.

## 5. Fetal and Neonatal Outcomes

The health of the mother, the growing fetus, and the newborn are all significantly impacted by maternal obesity. The effects of maternal obesity on fetal development and the ensuing immediate and long-term health effects on neonates are examined in this section. Obesity in mothers changes the intrauterine environment, which impacts fetal development and can lead to problems such as congenital defects, metabolic programming changes, and macrosomia. Understanding the health of newborns, as well as the long-term dangers, such as metabolic and cardiovascular disorders, for children born to obese women depends on these data [10].

### 5.1. Impact on Fetal Development

Fetal growth and development are significantly impacted by maternal obesity, which poses serious challenges during gestation. Obese pregnant women are at higher risk for stillbirth, abnormal growth trajectories, and fetal structural anomalies. According to Flenady et al. [3], maternal obesity significantly increases stillbirth risk by impairing placental function and reducing oxygen delivery to the fetus. More recent studies confirm that placental inflammation, oxidative stress, and altered lipid metabolism further compromise fetal well-being [64,65]. These findings emphasize the critical role of maternal health in fetal survival and development.

Maternal obesity is also associated with asymmetric fetal growth, notably, macrosomia and intrauterine growth restriction (IUGR). Macrosomia, defined as birth weight > 4000–4500 g, increases the risk of birth trauma and complications such as shoulder dystocia [66]. Excess nutrient and lipid transfer across the placenta alters fetal metabolic programming, affecting the liver, brain, and adipose tissue and predisposing to long-term cardiometabolic disorders [67,68]. This dual effect of both overgrowth and developmental disruption highlights the complexity of fetal outcomes in obese pregnancies [69].

In addition, maternal obesity is a well-established risk factor for both spontaneous and medically indicated preterm birth. This is especially evident in pregnancies complicated by gestational diabetes, preeclampsia, and chronic inflammation. Obesity-associated inflammatory cytokines and metabolic disturbances impair uterine quiescence and myometrial contractility, contributing to premature labor onset [2,45,70]. Placental dysfunction and premature rupture of membranes (PROM) are also more frequent due to increased oxidative and inflammatory stress [71,72].

Mechanistically, endocrine imbalances—including elevated leptin and reduced adiponectin—alter uterine tone and labor readiness. Animal studies support that maternal high-fat diets and obesity induce hypothalamic reprogramming, epigenetic alterations, and long-term neuroendocrine dysregulation in offspring [64,72,73,74].

In summary, maternal obesity exerts multifaceted stress on the fetus, increasing the risk of impaired growth, preterm birth, stillbirth, and lifelong metabolic disease. These findings reinforce the importance of targeted maternal health interventions to optimize fetal outcomes and reduce neonatal complications.

### 5.2. Neonatal Health at Birth and Long-Term Implications

Maternal obesity is highly associated with several short- and long-term neonatal issues. One of the most obvious effects is fetal macrosomia, which is defined by high birth weight and frequently leads to delivery problems such as shoulder dystocia and birth trauma. According to some hypotheses, the altered intrauterine environment caused by maternal obesity has a major effect on the newborn’s health and may result in metabolic and developmental problems later in life [75].

Beyond metabolic programming, maternal obesity also increases the risk of perinatal ischemic stroke (PIS), a severe neurological complication resulting from reduced cerebral blood flow or vascular occlusion in the perinatal period. Studies suggest that maternal obesity contributes to fetal vascular dysfunction through multiple mechanisms, including endothelial inflammation, hypercoagulability, and placental hypoxia [37]. Obese pregnancies are associated with increased fibrin deposition and thrombotic activity, predisposing the fetus to cerebrovascular events. Furthermore, maternal hyperlipidemia and insulin resistance have been implicated in altering placental perfusion, further increasing the risk of fetal hypoxic–ischemic injury [76].

A key mechanism linking maternal obesity to perinatal stroke involves pro-inflammatory cytokines and oxidative stress, which contribute to endothelial damage and blood–brain barrier dysfunction in the fetus [77]. Obese mothers exhibit elevated levels of IL-6, TNF-α, and ROS, which impair fetal vascular homeostasis and may trigger ischemic events in the developing brain. Additionally, maternal obesity-induced epigenetic modifications may exacerbate fetal cerebrovascular vulnerability, increasing the likelihood of long-term neurological impairments such as cerebral palsy, motor deficits, and cognitive dysfunction [76].

According to a new study by Hoffman and colleagues (2021), children of obese mothers are more likely to acquire metabolic syndrome when they become older [78]. Among these are the propensity for obesity, cardiovascular disorders, and insulin resistance [78]. According to the theory of developmental programming, unfavorable intrauterine circumstances—the two most significant of which are the mother’s excessive weight and disruptions in nutrition transport—can permanently affect the offspring’s metabolic health.

Further evidence of an intergenerational cycle of obesity is provided by Langley-Evans et al. (2022), who show that children of obese mothers are more likely to experience rapid postnatal weight gain and excessive adiposity, which set the stage for long-term metabolic disorders [58]. Fast postnatal weight increase and excessive fat buildup are more likely to occur in children who experienced an obesogenic intrauterine environment during critical perinatal periods, setting the stage for metabolic disorders. This indicates that children’s obesity is becoming even more common in this group, which will have an impact on their immediate health, as well as putting them at risk for several non-communicable diseases from adolescence into late adulthood, including type 2 diabetes and hypertension.

These findings emphasize the critical need for early interventions targeting maternal metabolic health to reduce neonatal risks and break the intergenerational transmission of obesity and related disorders [79]. Prenatal screening for vascular dysfunction, inflammatory markers, and metabolic abnormalities in obese pregnancies may provide an opportunity for early identification and targeted management of at-risk neonates. Additionally, promoting healthy maternal weight gain, improved glycemic control, and anti-inflammatory interventions may mitigate the risks of perinatal ischemic stroke and other long-term complications in offspring.

## 6. Maternal Progress and Recovery

The postpartum period is a critical time for maternal health, and recovery from obesity is especially challenging for obese mothers. Obesity in mothers frequently affects wellness and complicates the physical and emotional healing process after giving birth. This section will offer a chance to discuss the challenges that obese mothers may face during their postpartum recovery, including physical healing and mobilization problems, as well as the variables that lead to inadequate breastfeeding. The following barriers must be identified to enhance the health of mothers and their infants [80].

### 6.1. Postpartum Recovery Challenges

During the postpartum period, obese women often encounter several challenges, which exacerbate problems and delay healing. Maternal obesity has been associated with poor wound healing and elevated inflammation. An increased risk of postpartum infectious disorders further distinguishes these diseases and complicates the recovery process. Obese women have a harder time with physical rehabilitation because they are more likely to have various mobility problems, become tired fast, and have trouble managing daily activities after giving birth [36].

In addition to postpartum complications, maternal obesity is increasingly recognized as a significant risk factor for recurrent spontaneous abortion (RSA). Obese women are more likely to experience pregnancy loss due to dysregulated inflammatory responses, impaired endometrial receptivity, and metabolic disturbances [58]. Studies have demonstrated that excessive adiposity contributes to a pro-inflammatory intrauterine environment, characterized by elevated levels of TNF-α, IL-6, and CRP, which negatively affect embryo implantation and placental development [81]. Moreover, obesity-induced oxidative stress and mitochondrial dysfunction can lead to poor oocyte quality and chromosomal abnormalities, further increasing the risk of miscarriage [30].

Another major contributing factor to RSA in obese women is insulin resistance and altered glucose metabolism, which are commonly observed in women with polycystic ovary syndrome (PCOS) and GDM. Elevated maternal insulin and glucose levels create a hostile uterine environment that disrupts trophoblast invasion and impairs angiogenesis, essential for successful implantation and placental function [82]. Furthermore, obesity has been linked to hormonal imbalances, including reduced progesterone levels, which are crucial for maintaining pregnancy during the first trimester. Insufficient progesterone production in obese women has been associated with defective decidualization and an increased risk of early pregnancy loss [58].

Overweight mothers often experience significant long-term health issues during the postpartum phase. A high pre-pregnancy BMI considerably raises a woman’s risk of gaining weight during pregnancy and of developing chronic illnesses such as type 2 diabetes and cardiovascular disease. Mothers’ quality of life may be affected by the long-term health effects linked to a high pre-pregnancy BMI. This suggests that certain postpartum therapies would be essential for assisting mothers in their recuperation and lowering their overall risk of developing health issues in the future [83].

### 6.2. Breastfeeding Challenges

Breastfeeding has physical, physiological, and psychological difficulties, making it difficult to practice in obese individuals. According to an estimated figure, mothers who are obese are less likely than mothers of normal weight to start nursing or to continue breastfeeding for shorter periods [53]. Inequalities in breastfeeding are often brought about by delayed lactation initiation, difficulties with positioning, difficulties with latching, and a lack of confidence in one’s ability to breastfeed. The health of the mother is affected, the benefits of breastfeeding for the infants are diminished, and the need for tailored care and interventions to improve breastfeeding outcomes for obese mothers is highlighted.

### 6.3. Public Health Implications and Clinical Recommendations

Medical professionals and public health officials are being urged to take the lead because of the obvious negative impacts that maternal obesity has on the health of both the mother and the unborn child. To address the problems and potentially lessen the risks associated with pregnancy obesity, a range of clinical guidelines and preventative actions are required. The next section explores the role of prevention, from lifestyle modifications to personalized prenatal care, and examines the regulations that guide decisions on the optimal timing and method of delivery. Clinical guidelines and effective public health strategies can improve the health of mothers and their offspring by reducing the negative effects of maternal obesity [11].

## 7. Preventive Strategies and Recommendations

One important strategy that will lessen unfavorable outcomes for both the mother and the fetus is the prevention of obesity during pregnancy. To improve pregnancy outcomes and lower the risks of obesity-related problems, many public health sessions implement healthy lifestyle interventions both before and during pregnancy. 

Food, exercise, and behavioral support for weight management should be the focus of public health interventions for expectant mothers. According to Poston and colleagues (2016), these could lower the chances of preeclampsia, gestational diabetes problems, and excessive GWG during pregnancy. Healthy pregnancy trajectories for obese women are based on structured exercise regimens that incorporate motivational counseling and appropriate nutritional advice [20].

### 7.1. Lifestyle Modifications for Weight Management

According to Langley-Evans and associates, reducing the hazards associated with obesity during pregnancy requires significant lifestyle changes both before and during pregnancy [58]. According to their recommendations, healthcare professionals should inform expectant mothers about proper diet, exercise, and weight control. Customized, culturally relevant, and adaptable interventions are necessary to improve compliance and guarantee optimal outcomes for both the mother and the fetus.

### 7.2. Recommendations for Clinicians and Healthcare Providers

Following evidence-based standards, healthcare providers should monitor and assess gestational weight growth and incorporate weight management counseling into routine prenatal care. Pregnancy-related obesity can be effectively managed with the help of multidisciplinary preventive methods that involve a dietician, obstetrician, and physical activity specialist. Preventive measures require community support, public health campaigns, and healthcare experts working together. Early and consistent care can enhance the health of mothers and newborns while reducing the dangers associated with obesity [84].

### 7.3. Policies on Timing and Mode of Delivery

In the case of obese pregnant women, effective delivery planning includes determining the best time and method of delivery for the mother and the unborn child. Clear guidelines are essential for healthcare professionals when making decisions, given the heightened risks associated with maternal obesity. The research underscores the importance of planning individual deliveries, highlighting that decisions regarding elective or induced births must account for the specific risks associated with obesity, such as an increased likelihood of cesarean sections and prolonged labor [85]. These recommendations aim to minimize challenges for both the mother and newborn while carefully balancing risks and benefits.

Wen and colleagues provide further clarification on the criteria for determining the mode of delivery, noting that scheduled cesarean sections may be necessary in certain situations to mitigate the risks of complications from difficult labor or emergency cesarean deliveries [86]. The guidelines stress that healthcare professionals should consider the mother’s health, her history of previous deliveries, and the well-being of the unborn child when determining the safest delivery method to improve outcomes and reduce risks associated with maternal obesity during childbirth; proactive planning, patient education, and individualized assessment are essential [87].

According to the FIGO recommendations [84], maternal obesity represents a significant consideration when planning delivery, often necessitating individualized risk assessment for cesarean indications.

### 7.4. Postpartum Support for Weight Management and Mental Health

For obese women, postpartum care must fully address the psychological toll of childbirth and weight-related health issues, in addition to the physical recuperation. A number of postpartum issues, such as delayed wound healing, heightened vulnerability to infections, postoperative complications, and breastfeeding difficulties, are known to be associated with maternal obesity. For example, women with higher BMI are more likely to have wound infections from cesarean sections, which are linked to systemic inflammation, prolonged operating times, and inadequate tissue perfusion [88,89]. These issues may exacerbate psychological distress at a time when people are already at risk by causing chronic physical discomfort and reinforcing a negative body image. The effectiveness of structured postpartum lifestyle interventions, such as physical activity and nutritional counseling, in reducing these risks has been supported by recent data.

The effectiveness of structured postpartum lifestyle interventions has been highlighted in recent research, especially those that combine behavioral therapy, physical activity guidance, and nutritional counseling. Postpartum women who engaged in multi-component lifestyle programs saw notable improvements in their weight loss, metabolic health, and mental well-being, according to a systematic review by O’Reilly et al. Crucially, these treatments can also lower the long-term risk of comorbidities associated with obesity, such as cardiovascular disease and type 2 diabetes [90].

Mobile health (mHealth) platforms have gained traction to address access and adherence issues, particularly among women who are managing newborn care, fatigue, and socioeconomic constraints [72]. These digital interventions, which frequently include goal-setting, real-time feedback, and remote coaching, provide flexibility and personalization. According to studies, mHealth tools improved postpartum women’s engagement and maintained behavior change, especially for those from marginalized communities. Additionally, mobile interventions facilitate continuity of care during a time when in-person clinical follow-up may be irregular [91,92].

Obesity has an equally significant psychological impact during the postpartum phase. Obesity and perinatal mood disorders, such as anxiety and postpartum depression, have been linked in both directions [93]. Chronic low-grade inflammation is one of the biological mechanisms that partially mediate this relationship. In postpartum women who are obese, elevated pro-inflammatory markers like CRP and IL-6 have been repeatedly associated with depressive symptoms [66,73]. Negative self-image, social stigma, and the strain of managing weight-related health issues all increase the likelihood of mental health decline.

Recent studies suggest new treatment approaches that target the gut–brain axis, such as omega-3 fatty acids, prebiotics, and probiotic supplements. These interventions may provide supplemental benefits in postpartum mental health care because they have demonstrated promise in lowering systemic inflammation and elevating mood [94,95]. Peer support groups, cognitive behavioral therapy, and mindfulness-based stress reduction have also shown promise in treating depression symptoms in obese postpartum women.

## 8. Discussion

The results of this thorough analysis show that maternal obesity significantly changes the physiological environment of pregnancy via a number of interrelated pathways. Due mainly to complications like preeclampsia and GDM [29], our analysis demonstrates that pregnant women who are obese have significantly higher rates of medically indicated preterm birth (OR 1.5–2.3) [36]. Adipose tissue secretes pro-inflammatory cytokines (TNF-α, IL-6), which hinder placental angiogenesis and function, suggesting that chronic low-grade inflammation is the mechanism underlying this increased risk [23,96]. Because of the hostile intrauterine environment caused by these molecular changes, early delivery is frequently required to safeguard the health of both the mother and the fetus.

The convergence of several obesity-related issues is reflected in the significantly higher cesarean delivery rate (50–60% vs. 20–30% in normal BMI pregnancies) [71]. Fetal macrosomia, which affects 15–25% of obese pregnancies [75], along with labor dystocia brought on by decreased uterine contractility [57], makes vaginal delivery extremely risky. Crucially, obese women are at a two-fold increased risk of postpartum hemorrhage [80] and a three-fold increased risk of surgical site infections [63]; so, the decision to have a cesarean delivery carries extra considerations. According to recent data, protocolized care bundles that include negative-pressure wound therapy and preoperative chlorhexidine bathing may lower infection rates in this population by as much as 40% [62].

Our review focuses on the long-term postpartum difficulties that obese women encounter after giving birth. Adipose tissue hypoxia and chronic inflammation increase the risk of wound healing complications [27], and the risk of venous thrombosis stays high for up to 12 weeks after giving birth [71]. The breastfeeding disparities are perhaps the most concerning; obese women have lower breastfeeding continuation rates at 6 months (32% vs. 54%) [5] and delayed lactogenesis II (onset by 72 h in only 68% vs. 90% in women with normal BMI) [53]. These results highlight the necessity of long-term postpartum care plans designed specifically for this high-risk group.

By increasing food transfer to the fetus through the upregulation of nutritional transporters like FATP and GLUT1, maternal obesity also has an impact on placental function. Obesity also affects uterine contractility and the progression of labor. Decreased expression of gap junction proteins, such as connexin 43 and oxytocin receptors, interferes with coordinated uterine contractions, increasing the risk of cesarean delivery and extending labor. The difficulties obese mothers encounter during labor and delivery are highlighted by these molecular alterations [23]. Additionally, postpartum recovery is impacted by the molecular effects of fat. In addition to hindering the healing of wounds, particularly following cesarean sections, delayed tissue regeneration and increased inflammation can also disrupt hormonal balance, making nursing more difficult.

Maternal obesity may have the most extensive effects on developmental programming. Numerous studies now show that maternal metabolic dysfunction causes epigenetic changes in children, especially through DNA methylation of genes that control energy metabolism (e.g., leptin, PPAR-γ) [25,97]. The three-to-four-times higher risk of childhood obesity [69] and the earlier onset of metabolic syndrome in children of obese mothers [10] can be explained by these changes. Mechanistic insights from diet-induced obesity in animal models have demonstrated that maternal hyperglycemia and hyperlipidemia independently affect the development of fetal pancreatic β cells and the regulation of hypothalamic appetite [14,78].

Our analysis revealed a number of important knowledge gaps. First, while many studies show a higher risk of childhood obesity, few evaluate adult-onset cardiovascular disease by tracking offspring past adolescence [98]. Second, despite mounting evidence that preconception interventions may be more successful in lowering adverse outcomes, the majority of intervention trials concentrate on GWG management [69]. Third, even though obesity is rapidly increasing in low- and middle-income countries, where it often coexists with undernutrition, the great majority of research is conducted in high-income countries [4].

These findings have significant clinical and public health implications. Our review supports the following: (a) universal early GDM screening (<20 weeks) for pregnant women who are obese at the practice level [99]; (b) women with a BMI ≥ 40 and other risk factors may be given consideration for a planned cesarean delivery [84]; (c) giving high-risk obese women extended thromboprophylaxis for six weeks after giving birth [100]. Our review also supports (a) the inclusion of preconception counseling in primary care for obese women of reproductive age at the policy level [60], (b) the creation of uniform obstetric procedures tailored to obesity [87], and (c) the funding of long-term research monitoring metabolic impacts across generations [101].

Despite offering a thorough synthesis of the available data, this review has some limitations that should be noted. Direct comparisons are made more difficult by the variation in BMI classification among studies, and the majority of the included research uses observational designs rather than interventional trials. Furthermore, the relative contributions of maternal obesity and excessive GWG are still unclear and need more research.

In order to promote recovery and enhance maternal health outcomes, these postpartum issues highlight the necessity of customized care. The results indicate that the molecular mechanisms underlying maternal obesity should be the focus of targeted treatments. Interventions such as anti-inflammatory medications, improved insulin sensitivity management, and dietary therapy that reduces oxidative stress may improve outcomes for both mother and child. Public health campaigns should prioritize evidence-based clinical recommendations for postpartum care and delivery planning, lifestyle modifications, and preconception counseling. The molecular effects of maternal obesity on insulin resistance, inflammation, placental function, and uterine contractility have a substantial influence on the course of pregnancy. A more thorough understanding of these pathways is necessary to create effective plans and regulations to reduce health risks for women and children [73].

## 9. Research Gaps and Future Directions

Although there has been a great deal of progress in understanding maternal obesity and its impact on pregnancy outcomes, there are still several information gaps that need further clarity. According to Catalano and colleagues, more comprehensive research is needed to understand the underlying mechanisms via which maternal obesity impacts maternal and fetal health outcomes [36]. There is a dearth of longitudinal research on the effects of pregnancy-related interventions, such as dietary changes or physical activity, on lowering the risks of obesity. The long-term health impacts of such treatments on mothers and children must be further studied to develop effective prevention strategies.

Poston and colleagues also addressed the shortcomings in the current literature that call for the implementation of genuinely rigorous randomized controlled trials to assess the effectiveness of interventions to improve poor outcomes in obese pregnant women. They argue that more research on larger populations is needed to enhance generalizability, as most current studies focus on small groups. Research on the psychological elements that affect health behaviors in the case of obese pregnant women is also necessary, as is research explaining how education among healthcare workers improves results [20].

Future investigations must understand the relationship between genetic factors affecting fetal development and maternal obesity. Similarly, further research is needed to determine how social determinants of health are used to predict differences in pregnancy outcomes among populations of obese pregnant women. The improvement of clinical practice and the development of policies that can best help overweight women during pregnancy and even after the gestation period are dependent on these knowledge gaps.

## 10. Conclusions

Maternal obesity significantly impacts pregnancy outcomes, including birth timing and method, maternal recovery, and neonatal health. It raises the possibility of issues that may damage the health of the mother and the unborn child, including gestational diabetes, preeclampsia, cesarean birth, and difficult labor progression. Furthermore, maternal obesity raises the risk of neonatal problems like macrosomia, increased neonatal intensive care unit admissions, and long-term health issues like metabolic syndrome and juvenile obesity. Targeted interventions, such as updated healthcare standards, prenatal counseling, and lifestyle adjustments, are desperately needed, given the increased prevalence of maternal obesity. Addressing these issues through comprehensive policies and further research is essential to improve maternal and newborn health outcomes and reduce associated risks.

## Figures and Tables

**Table 1:** Molecular mechanisms of maternal obesity and its impact on pregnancy. This table summarizes the key molecular mechanisms of maternal obesity, highlighting their primary regulators, effects on pregnancy, and associated outcomes.

Molecular Mechanism	Key Regulators	Impact on Pregnancy	Outcome
Chronic Inflammation	TNF-a, IL-6, CRP	Endothelial dysfunction, Oxidative stress, Reduced NO generation	Preeclampsia, GDM
Hormonal Dysregulation	Leptin, adiponectin	Appetite dysregulation, Impaired glucose metabolism	Excessive GWG, GDM
Placental Dysfunction	GLUT1, FATP, ROS	Altered nutrient transport, Trophoblast dysfunction	Macrosomia, Fetal growth restriction, Stillbirth
Epigenetic Modifications	DNA methylation, histone modifications, miRNAs	Altered gene expression in metabolic pathways	Long-term risks, Childhood obesity, Type 2 diabetes
Uterine Contractility and Labor	Oxytocin receptors, connexin 43	Reduced contractility, Delayed labor onset	Prolonged labor, Cesarean delivery
Postpartum Recovery Challenges	MMPs, macrophages, prolactin	Impaired wound healing, Reduced lactation	Delayed recovery, Breastfeeding difficulties

## Data Availability

No new data were created or analyzed in this study.

## Abbreviations

The following abbreviations are used in this manuscript:

GDM	Gestational Diabetes Mellitus
NAFLD	Non-Alcoholic Fatty Liver Disease
GWG	Gestational Weight Gain
BMI	Body Mass Index
WHO	World Health Organization
TNF-α	Tumor Necrosis Factor-alpha
IL-6	Interleukin-6
CRP	C-Reactive Protein
ROS	Reactive Oxygen Species
IUGR	Intrauterine Growth Restriction
RAS	Renin–Angiotensin System
PIH	Pregnancy-Induced Hypertension
VBAC	Vaginal Birth After Cesarean
LPS	Lipopolysaccharide
FATP	Fatty Acid Transport Protein
GLUT1	Glucose Transporter 1
IRS-1	Insulin Receptor Substrate-1
Ang II	Angiotensin II
LDL	Low-Density Lipoprotein
VLDL	Very-Low-Density Lipoprotein
TGs	Triglycerides
FFAs	Free Fatty Acids
MMP	Matrix Metalloproteinase
PROM	Premature Rupture of Membranes
PIS	Perinatal Ischemic Stroke
RSA	Recurrent Spontaneous Abortion
PCOS	Polycystic Ovary Syndrome
SSIs	Surgical Site Infections
DVT	Deep Vein Thrombosis
mHealth	Mobile Health
PPAR-γ	Peroxisome Proliferator-Activated Receptor Gamma
NO	Nitric Oxide
PPROM	Preterm Premature Rupture of Membranes
FIGO	International Federation of Gynecology and Obstetrics
